# Magnetic resonance imaging: dynamic contrast enhancement and diffusion-weighted imaging to identify malignant cervical lymph nodes

**DOI:** 10.1590/0100-3984.2017.0005

**Published:** 2018

**Authors:** Murilo Bicudo Cintra, Hilton Ricz, Mahmood F. Mafee, Antonio Carlos dos Santos

**Affiliations:** 1 MD, PhD, Head and Neck Radiology, Radiology Division, Department of Internal Medicine, Faculdade de Medicina de Ribeirão Preto da Universidade de São Paulo (FMRP-USP), Ribeirão Preto, SP, Brazil.; 2 MD, PhD, Professor of Head and Neck Surgery, Faculdade de Medicina de Ribeirão Preto da Universidade de São Paulo (FMRP-USP), Ribeirão Preto, SP, Brazil.; 3 MD, FACR, University of California San Diego (UC San Diego) Health System in La Jolla, San Diego, CA, USA.; 4 MD, PhD, Professor of Neuroradiology, Faculdade de Medicina de Ribeirão Preto da Universidade de São Paulo (FMRP-USP), Ribeirão Preto, SP, Brazil.

**Keywords:** Lymph nodes/diagnostic imaging, Lymphatic metastasis/diagnostic imaging, Magnetic resonance imaging/methods, Diffusion magnetic resonance imaging, Linfonodos/diagnóstico por imagem, Metástase linfática/diagnóstico por imagem, Ressonância magnética/métodos, Difusão por ressonância magnética

## Abstract

**Objective:**

To examine the potential of two magnetic resonance imaging (MRI) techniques-dynamic contrast enhancement (DCE) and diffusion-weighted imaging (DWI)-for the detection of malignant cervical lymph nodes.

**Materials and Methods:**

Using DCE and DWI, we evaluated 33 cervical lymph nodes. For the DCE technique, the maximum relative enhancement, relative enhancement, time to peak enhancement, wash-in rate, wash-out rate, brevity of enhancement, and area under the curve were calculated from a semi-quantitative analysis. For the DWI technique, apparent diffusion coefficients (ADCs) were acquired in the region of interest of each lymph node. Cystic or necrotic parts were excluded. All patients underwent neck dissection or node biopsy. Imaging results were correlated with the histopathological findings. None of the patients underwent neoadjuvant treatment before neck dissection.

**Results:**

Relative enhancement, maximum relative enhancement, and the wash-in rate were significantly higher in malignant lymph nodes than in benign lymph nodes (*p* < 0.009; *p* < 0.05; and *p* < 0.03, respectively). The time to peak enhancement was significantly shorter in the malignant lymph nodes (*p* < 0.02). In the multivariate analysis, the variables identified as being the most capable of distinguishing between benign and malignant lymph nodes were time to peak enhancement (sensitivity, 73.7%; specificity, 69.2%) and relative enhancement (sensitivity, 89.2%; specificity, 69.2%).

**Conclusion:**

Although DCE was able to differentiate between benign and malignant lymph nodes, there is still no consensus regarding the use of a semi-quantitative analysis, which is difficult to apply in a clinical setting. Low ADCs can predict metastatic disease, although inflammatory processes might lead to false-positive results.

## Abstract (pt)

**Objetivo:**

Examinar o potencial das imagens de contraste dinâmico (DCE-MRI) e difusão (DW-MRI) em ressonância magnética na detecção de linfonodos cervicais malignos.

**Materiais e Métodos:**

Foram realizadas DCE-MRI e DW-MRI em 33 linfonodos cervicais. Os valores de realce relativo máximo, realce relativo, tempo de pico, taxa de realce e lavagem, brevidade do realce e área sob a curva foram avaliados pela análise semiquantitativa (DCE-MRI). Os coeficientes de difusão aparente na DW-MRI foram obtidos na área de interesse. Foram excluídas partes císticas ou necróticas dos nódulos. Todos os pacientes foram submetidos a dissecção cervical ou a biópsia. Os resultados de imagem foram correlacionados com os achados patológicos. Nenhum paciente foi submetido a tratamento neoadjuvante antes da dissecção do pescoço.

**Resultados:**

Realce relativo, realce relativo máximo e taxa de realce aumentaram nos nódulos malignos (*p* < 0,009, *p* < 0,05 e *p* < 0,03, respectivamente). O tempo de pico foi reduzido nos nódulos malignos (*p* < 0,02). A análise multivariada identificou tempo de pico (sensibilidade, 73,7%; especificidade, 69,2%) e realce relativo (sensibilidade, 89,2%; especificidade, 69,2%) como variáveis capazes de distinguir os nódulos benignos e malignos.

**Conclusão:**

Embora o DCE-MRI possa diferenciar os nódulos benignos e malignos, ainda não há consenso sobre a técnica de análise semiquantitativa, em razão de dificuldade de aplicação clínica. Valores baixos do coeficiente de difusão aparente podem predizer nódulo metastático, mas devem-se considerar também resultados falso-positivos, provavelmente secundários ao processo inflamatório.

## INTRODUCTION

Malignant cervical lymph nodes constitute a negative prognostic indicator in the treatment of head and neck cancer^(1-4)^. Therefore, early detection of malignant lymph nodes plays a crucial role in the clinical management and prognosis of head and neck cancer. The development of noninvasive imaging biomarkers for use in treatment planning has the potential to improve treatment strategies.

Anatomical imaging techniques such as ultrasound, contrast-enhanced computed tomography, and contrast-enhanced magnetic resonance imaging (MRI) are capable of detecting enlarged lymph nodes^(5-8)^, particularly in the cervical chains. However, such techniques are less sensitive for identifying malignancy in some cases^(9)^. Although ultrasound-guided fine needle aspiration biopsy of lymph nodes is capable of detecting malignancy, it is an invasive method that is operator-dependent and has a high rate of false-negative results^(10)^.

In this study, we propose a novel method of MRI incorporating anatomical and vascular information to improve the evaluation of lymph nodes. The addition of diffusion-weighted imaging (DWI)-to determine the apparent diffusion coefficient (ADC)-and dynamic contrast enhancement (DCE)-to quantify perfusion and vascularity-allows metastatic (malignant) lymph nodes to be distinguished from reactive (benign) lymph nodes. Our objective was to assess the ability of such methods to differentiate between benign and malignant lymph nodes. 

## MATERIALS AND METHODS

This was a prospective study in which patients under clinical suspicion of having head and neck cancer or patients with biopsy-confirmed cancer in the initial staging were recruited between August 2013 and October 2014. Patients who had undergone surgery of the head or neck, chemotherapy, or radiation therapy were excluded. All patients were screened for malignant cervical lymph nodes by an experienced head and neck neuroradiologist. The study was approved by the local institutional review board, and all participating patients gave written informed consent.

### Data acquisition

All MRI scans were acquired in a 3 T scanner (Achieva; Philips Medical Systems, Best, The Netherlands), with a phased-array neck coil. The MRI protocol included the following: *three-dimensional (3D) T1-weighted images*-repetition time/echo time (TR/TE) = 7.2/3.3 ms; field of view (FOV) = 240 mm; voxel size = 1.0 × 1.0 × 1.0 mm; slice thickness = 1 mm; and flip angle (FA) = 8°; *3D T2-weighted images*-TR/TE = 2500/304 ms; FOV = 240 mm; voxel size = 1.0 × 1.0 × 1.0 mm; section thickness = 1 mm; and FA = 90°; *DWI sequences*-TR/TE = 5174/55 ms; FOV = 222 mm; voxel size = 1.39 × 1.58 × 2.00 mm; section thickness = 2 mm; FA = 8°; directions = 4; and b values = 500 and 1000 s/mm^2^. In addition, we acquired DCE images using a 3D fast spoiled gradient-echo sequence with the following parameters: FOV = 300 mm; section thickness = 2 mm; gap = 1 mm; FA = 12°; TR/TE = 5.5/2.3 ms; voxel size = 0.9 × 0.99 × 2.0 mm; scan duration = 5 min. Using that protocol, we acquired non-contrast-enhanced images in 13 dynamic acquisitions. For contrast-enhanced images, patients received a single dose of gadodiamide (Gd-DTPA-BMA, Omniscan; Nycomed, Oslo, Norway) injected into the antecubital vein at a concentration of 0.1 mmol/kg body weight and at a rate of 2 mL/s, followed by a saline flush, both administered with a power injector (Spectris; Medrad, Indianola, PA, USA). Twelve dynamic acquisitions were performed during and after the injection.

### Imaging processing

**Figure 1:**
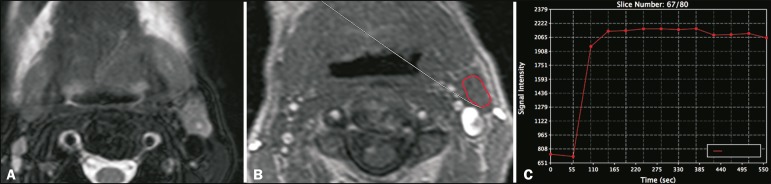
DCE MRI scan of a 72-year-old male patient with left oral tongue squamous cell carcinoma. A T2-weighted image (**A**) shows a stage IIa malignant lymph node, with a small necrotic center. The image on **B** shows the ROI (red outline) in the node during the DCE sequence. The image on **C** shows the time-signal intensity curve for the corresponding node.

Images were processed on a workstation (Philips Extended MR Workspace 2.6.3.5; Philips Medical Systems). Lymph nodes located in tumor drainage cervical chains were chosen, and a region of interest (ROI) was drawn on the solid portion of each node, for DWI and DCE. A head and neck radiologist with 5 years of experience delineated the ROIs, using T2-weighted, T1-weighted, or contrast-enhanced T1-weighted images. Necrotic, cystic, and hemorrhagic portions of the nodes were excluded. Single nodes and larger node masses were included. For DWI, the positioning of the ROI was determined by visual identification of the lowest signal on the ADC map^(11)^. For DCE acquisitions (Figure 1), time-signal intensity curves were generated for each lymph node ROI and the following parameters were evaluated: maximum relative enhancement (MRE); relative enhancement (RE); time to peak enhancement, hereafter simply time to peak (TTP); wash-in rate (WiR); wash-out rate (WoR); brevity of enhancement (BrevE); and area under the curve (AUC).

### Statistical analyses

All statistical analyses were performed with the IBM SPSS Statistics software package, version 22.0 for Windows (IBM Corporation, Armonk, NY, USA). The Shapiro-Wilk test showed that the data were not normally distributed. After correlation with histopathology, we analyzed two groups of data: malignant and benign lymph nodes. The Mann-Whitney U test was used in order to compare the benign and malignant groups. Values of *p* < 0.05 were considered significant. To identify further relationships among RE, TTP, WiR, and MRE, multivariate analysis (binary logistic regression) was applied. In that analysis, TTP and RE were the parameters found to be most capable of differentiating between malignant and benign nodes. We used a receiver operating characteristic (ROC) curve to determine the TTP and RE cut-off values for distinguishing malignant nodes from benign nodes.

## RESULTS

Our study sample comprised 19 patients (mean age, 55-68 years; 12 males and 7 females) with 33 lymph nodes (Table 1). The diameter of the lymph nodes ranged from 0.7 cm to 6.8 cm (mean, 2.2 cm). Thirteen (39.4%) of the lymph nodes were benign, and 20 (60.6%) were malignant. The malignant lesions were confirmed by histopathology following surgical removal in 25 (75%) of the nodes and by fine needle aspiration biopsy alone in 8 (25%).

**Table 1:** Demographic characteristics of the patients and histological diagnosis.

Patient	Gender	Age (years)	Diagnosis	Number of nodes
1	Male	40	Metastatic oropharyngeal SCC	1
2	Male	14	Metastatic nasopharyngeal carcinoma	1
3	Female	33	Submandibular neuroendocrine carcinoma	1
4	Female	42	Laryngeal SCC	2
5	Male	31	Lesion of cervical muscle/IgG4-related disease	2
6	Female	33	Inflammatory process	3
7	Male	91	Cutaneous SCC	1
8	Male	49	Nasopharyngeal carcinoma	2
9	Female	91	Frontal skin SCC	1
10	Male	68	Laryngeal SCC	2
11	Female	56	Inflammatory process	2
12	Male	81	Undifferentiated carcinoma	2
13	Male	12	Inflammatory process	2
14	Female	57	Pyriform sinus carcinoma	2
15	Male	82	Melanoma	3
16	Male	77	Undifferentiated carcinoma	1
17	Female	54	Inflammatory process	1
18	Male	63	Hypopharyngeal SCC	2
19	Male	75	Nonspecific inflammatory process	2

SCC, squamous cell carcinoma; IgG4, immunoglobulin G4.

Malignant and benign lymph nodes both showed low mean ADCs (0.786 ± 0.152 × 10^-3^ mm^2^/s and 0.790 ± 0.173 × 10^-3^ mm^2^/s, respectively). However, the difference was not statistically significant. No statistically significant differences were found among the ADC, WoR, BrevE, and AUC values in terms of their capacity to differentiate between malignant and benign lymph nodes. From the DCE images (Tables 2 and 3), we determined that the malignant lymph nodes presented significantly higher RE (*p* < 0.009), MRE (*p* < 0.05) and WiR (*p* < 0.03), whereas they presented significantly shorter TTP (*p* < 0.02). In the multivariate analysis, the differences between the values obtained for benign nodes and those obtained for malignant nodes remained significant for TTP and RE.

**Table 2:** DCE parameters in the malignant and benign groups.

		TTP (s)		RE (%)		MRE (%)		WiR (L/s)
Group	N	Median (SD)		Median (SD)		Median (SD)		Median (SD)
Malignant lymph nodes	19	141 (73)		97 (61)		598 (353)		21 (12)
Benign lymph nodes	13	207 (75)		38 (67)		392 (216)		13 (8)
*P-*value		0.02		0.009		0.05		0.03

SD, standard deviation.

**Table 3:** Main DCE parameters, by group.

Group	Values	RE (%)	MRE (SI)	TTP (s)	WiR (L/s)
	Mean	41.3	392.38	199.98	14.57
Benign lymph nodes	N	10	13	12	9
	SD	75.49	216.161	73.387	8.501
	Mean	95.67	594.74	146.47	21.15
Malignant lymph nodes	N	17	18	18	19
	SD	61.406	363.805	71.799	12.374
	Mean	75.53	509.88	167.88	19.03
Total	N	27	31	30	28
	SD	70.774	322.481	75.998	11.546

SI, signal intensity; SD, standard deviation.

**Figure 2:**
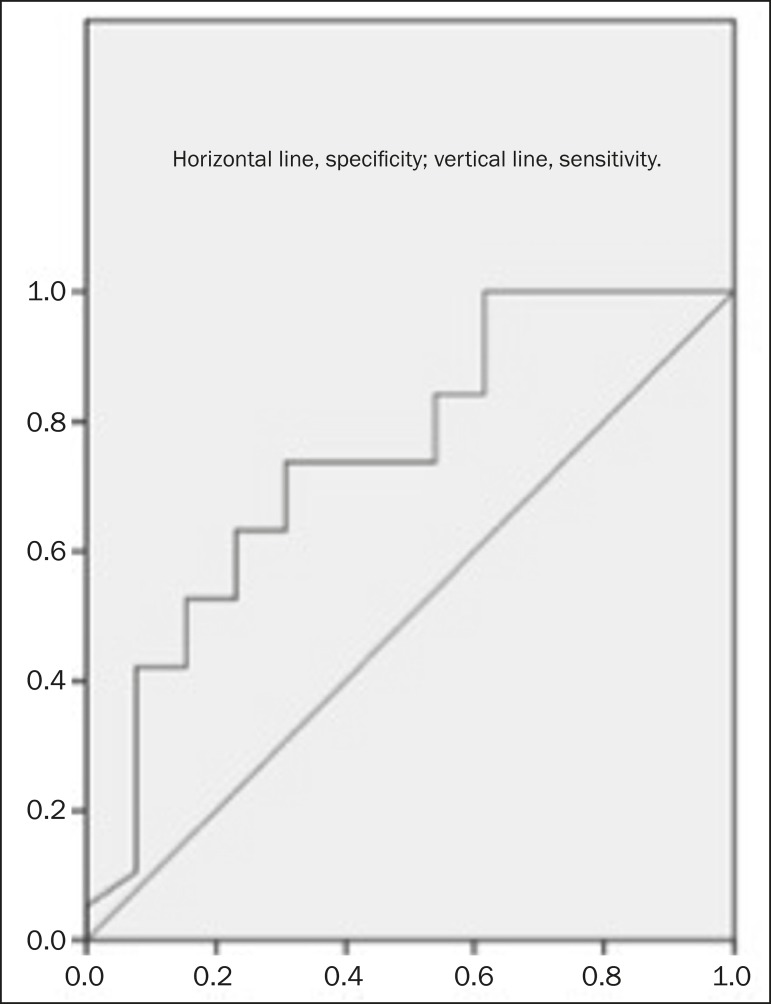
ROC curve for TTP.

**Figure 3:**
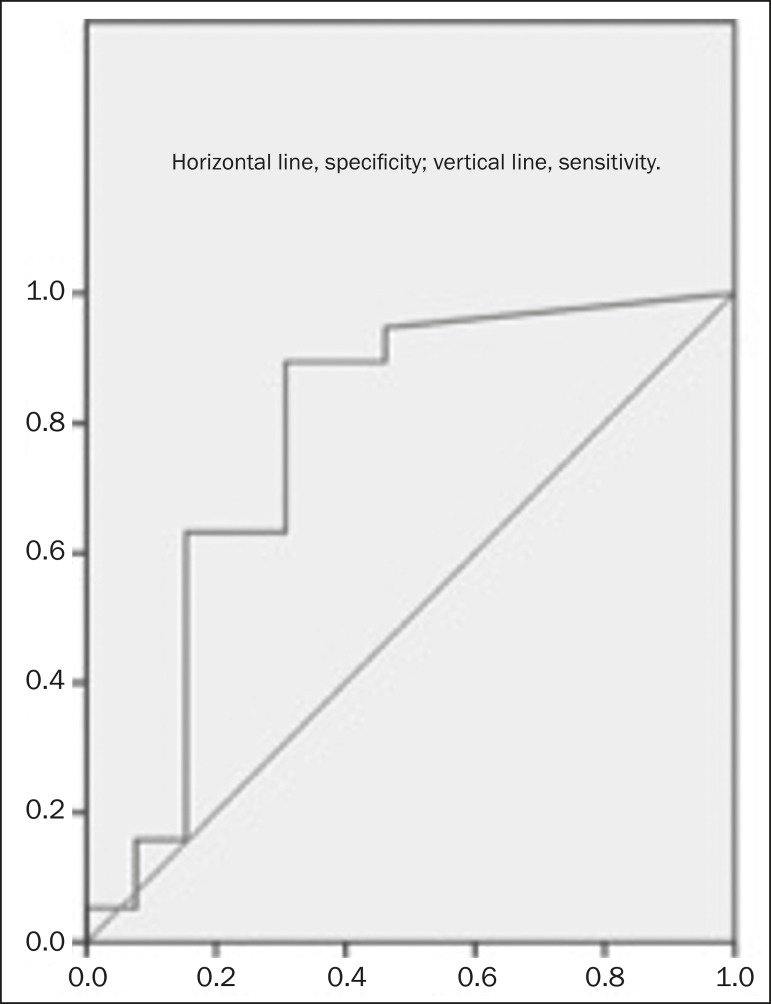
ROC curve for RE.

In the ROC curve analysis, the TTP cut-off value for malignant lymph node detection was 189.45 s. The TTP for the malignant nodes was significantly lower than was that determined for the benign nodes. The sensitivity and specificity of the TTP cut-off value to differentiate between benign and malignant lymph nodes were 73.7% and 69.2%, respectively (Figure 2). According to the ROC curve analysis, the RE cut-off value for malignant lymph node detection was 21.9%. The RE for the malignant nodes was significantly higher than was that determined for the benign nodes. The sensitivity and specificity of the RE cut-off value to differentiate between benign and malignant lymph nodes were 89.2% and 69.2%, respectively (Figure 3).

## DISCUSSION

No statistically significant differences were found among the ADC, WoR, BrevE, and AUC values in terms of their capacity to differentiate between malignant and benign lymph nodes. From the DCE images, we determined that the RE, MRE, and WiR were significantly higher in malignant lymph nodes, whereas the TTP was significantly shorter. The multivariate analysis showed that the TTP and RE differed significantly between benign and malignant nodes.

### DCE

Others studies involving DCE have suggested that it can be a useful tool to differentiate between benign and malignant tumors^(12)^, as well as between benign and malignant lymph nodes^(13)^. However, there have been few studies evaluating DCE parameters in metastatic disease of the head and neck. Fischbein et al.^(13)^ evaluated 68 lymph nodes and demonstrated significant differences between normal nodes and tumor-involved nodes, the latter showing longer TTPs, lower peak enhancement, a lower maximum slope, and a lower wash-out slope. In comparison with our study, that study employed different technical parameters for DCE image acquisition, including the initial presence of a contrast agent, the timing of the scan initiation, the duration of acquisition, the size of the standardized ROIs, and the partial volume effects in the ROI outlining, as well as demonstrating a different degree of interobserver variability. Those differences could account for the discrepancies between the results of the two studies.

### ADC

Although malignant and benign lymph nodes both showed low ADCs in the present study, the difference between the two was not statistically significant. Most of the data in the literature suggest that ADCs are lower in malignant lymph nodes. However, Sumi et al.^(14)^ found that ADCs were lower in malignant lymph nodes than in benign nodes. That unexpected result could be due to the fact that those authors included necrotic areas in the ROIs. Our finding that ADCs were lower in malignant nodes is consistent with the findings of Lee et al.^(15)^ and Holzapfel et al.^(16)^. However, as previously mentioned, we also found that the ADCs for benign nodes were similar to those for malignant nodes, with no statistical difference between the two. That finding could be related to the fact that many causes of cervical lymphadenopathy, including infectious disease, inflammatory/granulomatous disease, autoimmune disease, and neoplasia, result in lymph node hyperplasia with high cellularity. Another point is that the tumor microenvironment is largely orchestrated by inflammatory cells and participates in the neoplastic process, a processes that also results in increase cellularity secondary to hyperplasia due to lymph node reactions. Therefore, although the ADC can quantify changes in diffusion behavior, it cannot distinguish the cause of those changes^(17-19)^. In addition, in some neoplastic processes, other causes of cervical lymphadenopathy can mimic neoplasm on an ADC map. Currently, there is no consensus regarding the technical parameters for ADC acquisition, which limits the reproducibility and scalability of clinical studies.

Our findings indicate the potential of quantitative imaging to differentiate between malignant and benign cervical nodes during the investigation of metastatic disease prior to invasive procedures, potentially minimizing the use of such procedures. However, there is still a need for further studies, with larger patient samples, in order to confirm our findings.

Our study has some limitations. First, we evaluated a relatively small number of patients. In addition, the lack of standardization in the literature regarding the acquisition of DCE time-signal intensity curve parameters and ADCs, together with the inflammatory environment generated by the neoplastic process, could explain the low ADC values we found in benign lymph nodes. Furthermore, it is possible that artifacts occurred during DWI acquisition. The same radiologist performed all DWI measurements, in which we used single-shot echo-planar imaging, which is highly sensitive to static magnetic field (B0) heterogeneity, which produces nonlinear geometric distortion, primarily in the phase-encoding direction. Such artifacts become more severe at higher magnetic field strengths and can alter the ADC, potentially reducing the capability of the ADC to differentiate between malignant and benign lymph nodes.

## CONCLUSIONS

We conclude that perfusion MRI has the potential to identify malignant lymph nodes. However, because of technical differences across studies and the lack of a consensus in the literature, quantitative imaging still cannot replace or preclude the need for invasive methods for the diagnosis of malignant nodes.

The high cellularity of malignant lymph nodes results in a measurable decrease in their ADC, although other inflammatory processes cause high cellularity and can thus mimic malignant nodes. Additional studies with larger patient samples should be conducted. Further standardization of DWI and DCE techniques in different MRI scanners is fundamental to obtaining data that are reproducible and comparable across studies.
